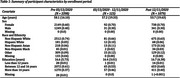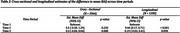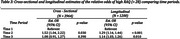# Research Attitudes Through the COVID‐19 Pandemic

**DOI:** 10.1002/alz70860_101401

**Published:** 2025-12-23

**Authors:** Sarah Schlund, Dan Hoang, Josh D Grill, Daniel L Gillen

**Affiliations:** ^1^ University of California, Irvine, Irvine, CA, USA; ^2^ The UC Irvine Institute for Memory Impairments and Neurological Disorders, Irvine, CA, USA; ^3^ The UC Irvine Institute for Alzheimer's Disease Research Center, Irvine, CA, USA

## Abstract

**Background:**

Increasing clinical research participation is crucial for biomedical advancement. The Research Attitudes Questionnaire (RAQ) is a validated instrument that predicts willingness to join trials and retention behaviors. The COVID‐19 pandemic offers a unique opportunity to assess how a public health emergency affects views on medical research. This study examines changes in research attitudes, measured by the RAQ, throughout the pandemic.

**Method:**

We conducted a longitudinal study of 4,600 adults who joined the UCI Consent‐To‐Contact (C2C) recruitment registry between 2016 and 2024 (Table 1). Research attitudes of C2C participants are evaluated annually using the RAQ. We a priori defined three phases of the COVID‐19 pandemic: T1 (before March 13, 2020, the date of declaration of a national emergency in the US), T2 (March 13 to December 11, 2020, the date of the first approvals of a COVID‐19 vaccine), and T3 (after December 11, 2020). A cross‐sectional analysis compared initial RAQ scores at time of enrollment into the registry for participants enrolling during each phase; a longitudinal analysis assessed changes in RAQ scores within individuals over time. Covariates included demographics, recruitment source, and chronic health condition status.

**Result:**

Estimates from the cross‐sectional and longitudinal models for mean total RAQ score are presented in Table 2. For the longitudinal analysis, mean within‐individual total RAQ score was significantly higher during T2 compared to T1 (Est. 0.44; 95% CI: 0.17, 0.71; *p* <0.001) followed by no significant difference between T3 and T1. Estimated odds ratios for the likelihood of having a high RAQ score (over 28) for cross‐sectional and longitudinal comparisons are shown in Table 3. Individuals enrolling during T2 were estimated to have a 52% increase in the odds of a high RAQ compared to those enrolling during T1 (adj. odds ratio (aOR)=1.52; 95% CI: 1.04, 2.22; *p* = 0.03). In the longitudinal analysis we estimated that the within‐individual odds of a high RAQ score were 31% higher during T2 when compared to T1 and (aOR=1.31; 95% CI: 1.16, 1.47; *p* <0.001).

**Conclusion:**

These findings suggest that awareness of urgent medical issues, such as the COVID‐19 pandemic, may enhance individuals' attitudes toward research.